# Adherence to Lower‑Risk Cannabis Use Guidelines among Canadian college students: a regression analysis

**DOI:** 10.24095/hpcdp.46.6.01

**Published:** 2026-06

**Authors:** Julia Pei, Laura B. Jones, Chris G. Richardson, Richard J. Munthali, Kristen L. Hudec, Angel Y. Wang, Lonna Munro, Daniel V. Vigo

**Affiliations:** 1 Department of Psychiatry, University of British Columbia, Vancouver, British Columbia, Canada; 2 School of Population and Public Health, University of British Columbia, Vancouver, British Columbia, Canada

**Keywords:** cannabis, substance-related disorders, students, adolescents

## Abstract

**Introduction:**

The objective of this study is to evaluate adherence to seven Canadian Lower-Risk Cannabis Use Guideline (LRCUG) recommendations among Canadian university students and identify subgroups of high-risk users.

**Methods:**

We analyzed survey data collected across four Canadian universities under the World Mental Health-International College Student (WMH-ICS) initiative. Seven of the ten 2017 LRCUG recommendations were evaluated. Zero-inflated Poisson models were employed to examine the sociodemographic correlates of (1) any lifetime cannabis use; and (2) the number of unmet LRCUG recommendations, conditional on lifetime use. Additionally, multivariable binary logistic regression models examined the sociodemographic correlates of adherence to individual recommendations.

**Results:**

Among the 27 236 respondents, the prevalence of lifetime cannabis use was 33.8%. Of the seven recommendations evaluated, “choosing lower-strength cannabis products” had the lowest adherence rate (29.0%), followed by “not smoking cannabis” (36.7%). “Not using synthetic cannabis” had the highest adherence rate (96.1%), followed by “delaying cannabis use until age 16” (91.2%). Men, non-heterosexual students, students living in shared housing, and domestic students were more likely to use cannabis and, among users, reported risky use. While White students were more likely to use cannabis, among users, many non-White student groups reported riskier use.

**Conclusion:**

Although most students did not use cannabis and many of the LRCUG recommendations had high rates of adherence, there were low rates of choosing lower-strength cannabis products and avoiding smoking cannabis among users. Study findings highlight specific recommendations and subpopulations to inform tailoring of future interventions targeting university students.

## Highlights

- One third of Canadian university students surveyed reported using cannabis in their lifetime.

- Optimistically, 91.2% of users reported delaying cannabis use until age 16 and 96.1% of users reported avoiding synthetic cannabis.

- Among users, the most commonly reported risky cannabis use behaviours were not choosing lower-strength cannabis products (71.0%) and smoking cannabis (63.3%).

- Riskier cannabis use was more common among men, non-heterosexual students, students living in shared housing, and domestic students.

- Further efforts are needed to promote literacy about lower-risk cannabis use among university students and increase the availability of lower-potency cannabis products. The adoption of a THC standard unit on labels should also be considered.

## Introduction

Despite decades of prohibition and criminal sanctions, Canada has among the highest rates of cannabis use in the world, with a lifetime prevalence of nearly 50% among Canadian adults.^1^^-^^3^ In 2018, the Canadian Government passed the *Cannabis Act* to legalize recreational (i.e. non-medical) cannabis use for adults (aged 18 years or older) with a goal of providing a quality-controlled supply of cannabis products, minimizing illicit cannabis-related activities, and reducing burden on the criminal justice system.^4^ According to the Canadian Substance Use Survey, 12-month cannabis use increased from 22% in 2018 (prelegalization) to 27% in 2022 (postlegalization).^5^

While recreational cannabis use is both legal and relatively common in Canada, the substance is associated with many adverse health outcomes such as respiratory and cardiovascular disorders, cognitive impairment, addiction, psychosis and schizophrenia.^6^^-^^10^ Further, the THC in cannabis can interfere with brain development, particularly within the frontal cortex. Given the human brain continues developing into the early 20s, adolescents and young adults may be particularly vulnerable to the adverse effects of cannabis use.^11^^,^^12^ Of note, a recent Ontario-based cohort study identified a 60% increase in psychosis incidence among people aged 14 to 20 between 1997 and 2023, aligning with increased rates of cannabis and other substance use.^13^

Many of these associations require further research, but the literature to date suggests that these adverse outcomes are typically concentrated among a small group of high-risk consumers, and can be substantially mitigated by informed behavioural choices.^14^ This knowledge led to the development of the Canadian Lower-Risk Cannabis Use Guidelines (LRCUG), which aim to serve as an evidence-based public health tool to guide Canadians towards safer consumption, similar to “Low Risk Drinking Guidelines” that have been used in alcohol public health initiatives.^15^

Originally published in 2011 and revised in 2017 in light of upcoming legalization, the LRCUG contain 10 recommendations on modifiable behavioural risk factors to reduce cannabis-associated harms.^15^^,^^16^ They advise that the only way to completely avoid risk is to not consume the substance, and recommend that users avoid early initiation of cannabis, high-strength and synthetic products, smoking cannabis, deep inhalation, daily/near-daily use, and driving or operating machinery after consumption. They also advise against use in high-risk populations and combining risk behaviours addressed in these guidelines. The LRCUG have been endorsed by many national organizations including the Public Health Agency of Canada, Canadian Medical Association, and Mental Health Commission of Canada.^17^ While these guidelines have been disseminated through knowledge translation materials such as brochures, posters and webinars since 2017, the effectiveness of these efforts remains unclear.

In response to an evolving body of scientific literature, the LRCUG were further updated in 2022.^8^ Grading of evidence and guideline structure were updated, and several new recommendations were added, including encouraging the use of legal and quality-controlled cannabis products, recommending against vaping, and cautioning against combining other psychoactive substances with cannabis use. While these updated recommendations provide important context in the interpretation of our findings, this study is based on examining adherence to the 2017 guidelines using data collected from 2020 to 2022.

Several studies have examined adherence to the LRCUG in general Canadian and American population samples and found varying levels of compliance across the different recommendations. Among those who consumed cannabis, the most common high-risk behaviours were smoking cannabis, use of high-strength products, daily cannabis use, and driving under the influence.^18^^,^^19^ While adherence to the 2017 guidelines has been examined in other studies, to our knowledge, no studies have examined adherence among university students. Due to their younger age, university students are particularly vulnerable to the adverse effects of cannabis and comprise a subpopulation where high-risk substance use behaviour is typically concentrated.^20^^,^^21^ As such, public health efforts addressing cannabis use in university students offer an opportunity for early intervention and prevention of high-risk behaviours.

This study examines the adherence to seven of the ten 2017 Lower-Risk Cannabis Use Guideline recommendations among students from four Canadian universities and identifies the sociodemographic characteristics of high-risk users to inform future knowledge translation efforts.

## Methods

### Study design

The World Health Organization (WHO) World Mental Health-International College Student (WMH-ICS) initiative uses validated screening instruments to generate estimates for a range of substance use and mental disorders.^22^ This study analyzed data collected through the repeated cross-sectional deployment of the WMH-ICS survey at four Canadian Universities: University of British Columbia (UBC), Simon Fraser University (SFU), McMaster University, and University of Toronto (UofT). The survey is based on the WHO World Mental Health-Composite International Diagnostic Interview (WHO WMH-CIDI).^23^

The survey is self-administered online using the Qualtrics survey platform.^24^ It was sent via email to a new group of 350 students at each institution weekly. Groups were generated via stratified random sampling (by gender, age, degree type and year, and international student status), and a rigorous follow-up strategy was employed to minimize non-responder bias, per a specified protocol described in a previous paper.^25^ This study contains 138 weeks of data dating from 9 February 2020, to 18 September 2022. Due to the staggered launch of the survey sites, data collection periods varied across sites. The survey’s adjusted response rate was 43.5% using the American Association for Public Opinion research weighted response rate 1 (RR1w) calculation for two-phase sample designs.^26^

### Outcome measures

In addition to the “abstain from use” recommendation, seven individual recommendations were operationalized: 1) having delayed cannabis use until at least the age of 16 years; 2) choose lower-strength cannabis products, such as those with a lower tetrahydrocannabinol (THC) content or a higher ratio of cannabidiol (CBD) to THC; 3) do not use synthetic cannabis products; 4) do not smoke cannabis; 5) do not inhale deeply or hold your breath when smoking cannabis; 6) if using cannabis, limit cannabis use to once a week or on weekends; and 7) do not drive a car or operate other machinery while under the influence (this assessment used the phrase “alcohol/other drug use” so was not specific to cannabis). As such, our outcome measures were lifetime cannabis use (representing the “abstain from use” recommendation) and the number of other unmet Canadian LRCUG recommendations. Of note, we assumed respondents who did not know the difference between THC and CBD did not choose lower-strength products, given most products on the market are high in THC. Based on the 2017 LRCUG, vaping was not categorized as smoking cannabis. The final recommendation to avoid combining high-risk behaviours was not operationalized as a separate guideline, since it was implicit in reporting the number of other unmet recommendations. The remaining guidelines were not operationalized. The cannabis survey questions are available upon request from the authors.

### Ethics approval

All procedures contributing to this work comply with the ethical standards of the relevant national and institutional committees on human experimentation and with the Helsinki Declaration of 1975, as revised in 2008. All procedures involving human subjects were approved by the Behavioural Research Ethics Board of the University of British Columbia (approval number H19-02538). Written informed consent was obtained from all participants before completing the WMH-ICS survey.

### Statistical analyses

Descriptive statistics were used to characterize the sample and adherence to individual recommendations. Zero-inflated Poisson models were then employed to examine the sociodemographic correlates of 1) any lifetime recreational cannabis use; and 2) the number of LRCUG recommendations currently unmet, conditional on lifetime recreational cannabis use. The models consisted of 1) logistic regression predicting the odds of lifetime cannabis use; and 2) Poisson regression predicting the count of recommendations unmet. Logistic regression coefficients were exponentiated to obtain odds ratios and Poisson regression coefficients were exponentiated to obtain incidence rate ratios for the number of unmet recommendations among lifetime non-medical cannabis users. Incidence rate ratios represented the relative incidence of LRCUG recommendation non-adherence. Separate univariable models for each sociodemographic predictor were run, as well as one multivariable model with all predictors included. To address potential issues of homoscedasticity, the vce(robust) option was used to obtain robust standard errors. Analyses were conducted using the zip command in Stata.^27^

Next, separate multivariable binary logistic regression models were used to examine the sociodemographic correlates of adherence to each of the seven LRCUG recommendation variables evaluated. As with the zero-inflated Poisson models, the vce(robust) option was used, and separate univariable logistic regressions were run prior to the multivariable logistic regression. The gender variable was collapsed into three categories (cisgender man, cisgender woman, other) for these analyses to avoid complete separation due to small sample sizes in the other gender categories. Bonferroni correction was used to adjust for multiple comparisons.

The sociodemographic predictors included in each of the models were gender, sexual orientation, race and ethnicity, housing type, international student status, and age.

Since lifetime cannabis use measures were used, age effects could not be meaningfully interpreted; the higher rates of cannabis use in older students could be attributable to their use in previous years. Therefore, while age was included as a covariate to control for possible confounding, age trends were not reported.

All analyses were conducted using Stata Version 16.1 for Mac, with a significance level of 5% (*p* < 0.05).^27^ Respondents with incomplete data were included in analyses if they had complete data for each of the outcome variables; missing values were categorized as “missing” for each covariate.

## Results

The study sample comprised 27 236 respondents. The majority of the respondents were cisgender women (62.6%), and the average age was 21.9 years (range: 18–25 years). The sample was relatively ethnically diverse (35.1% White, 24.6% East Asian, 14.3% South Asian, 0.7% First Nations, Inuit, or Métis, and 25.3% other visible minority).

The characteristics of the study sample, stratified by lifetime non-medical cannabis use, are presented in Table 1. Overall, 33.8% of students indicated lifetime cannabis use. There were significant differences in all characteristics evaluated (gender, sexual orientation, race and ethnicity, student type, housing type, and student status).

**Table 1:** Sample characteristics, stratified by lifetime non-medical cannabis use, WMH-ICS survey (N = 27 236)

**Characteristics**	**No lifetime non-medical cannabis use** **N (row %)**	**Lifetime non-medical cannabis use** **N (row %)**
**Overall**	18 036 (66.22%)	9 200 (33.78%)
**Gender**	*	*
Cisgender man	6 263 (66.83%)	3 108 (33.17%)
Cisgender woman	11 371 (66.76%)	5 661 (33.24%)
Transgender man	39 (53.42%)	34 (46.58%)
Transgender woman	28 (60.87%)	18 (39.13%)
Non-binary	223 (44.78%)	275 (55.22%)
Two-spirit	7 (38.89%)	11 (61.11%)
Other	87 (49.71%)	88 (50.29%)
**Sexual orientation**	*	*
Heterosexual	14 096 (70.02%)	6 036 (29.98%)
Gay or lesbian	498 (53.09%)	440 (46.91%)
Bisexual	1 205 (42.46%)	1 633 (57.54%)
Asexual	307 (77.72%)	88 (22.28%)
Questioning	893 (66.39%)	452 (33.61%)
Other	332 (51.00%)	319 (49.00%)
**Race and ethnicity**	*	*
White	4 588 (48.98%)	4 779 (51.02%)
First Nations, Inuit, or Métis	101 (52.33%)	92 (47.67%)
Hispanic or Latino	298 (60.20%)	197 (39.80%)
Black	371 (73.47%)	134 (26.53%)
Arab	364 (74.90%)	122 (25.10%)
East Asian (e.g. Chinese, Korean, Japanese)	5 573 (84.98%)	985 (15.02%)
Southeast Asian (e.g. Filipino, Vietnamese, Cambodian, Laotian, Thai)	570 (76.10%)	179 (23.90%)
South Asian (e.g. East Indian, Pakistani, Sri Lankan)	2 942 (77.12%)	873 (22.88%)
West Asian (e.g. Afghan, Iranian)	642 (76.34%)	199 (23.66%)
Other	497 (70.90%)	204 (29.10%)
Multiracial	1 688 (57.08%)	1 269 (42.92%)
**Student type**	*	*
Undergraduate	12 653 (68.70%)	5 765 (31.30%)
Graduate	4 095 (61.64%)	2 548 (38.36%)
Other	1 273 (58.99%)	885 (41.01%)
**Housing type**	*	*
With parents or other relatives	8 773 (72.58%)	3 315 (27.42%)
In their own home or apartment	4 493 (60.58%)	2 924 (39.42%)
In a university-owned or -operated residence or fraternity	1 760 (68.80%)	798 (31.20%)
In a shared house, apartment, or flat	2 754 (57.41%)	2 043 (42.59%)
Other	245 (67.49%)	118 (32.51%)
**Student status**	*	*
Domestic	14 549 (63.93%)	8 207 (36.07%)
International	3 487 (77.83%)	993 (22.17%)

**Abbreviation :** WMH-ICS, World Mental Health-International College Student.

**Notes:** Non-medical use is defined as use without a prescription or in larger doses than prescribed.

The extent of missing data varied across variables. Therefore, the Ns did not consistently sum to the total sample size of 8590 respondents.

Chi-square tests were conducted to test for differences in treatment rates across categories. All cell sizes were larger than 5.

* *p* ≤ 0.05.

### Overall Lower-Risk Cannabis Use Guideline adherence

The adherence rate for each of the seven LRCUG recommendations is presented in Table 2. The prevalence of lifetime recreational cannabis use was 33.8%. Among those who had used cannabis (N = 9200), “choosing lower-strength cannabis products” had the lowest adherence rate (29.0%), followed by “not smoking cannabis” (36.7%). “Not using synthetic cannabis” had the highest adherence rate (96.1%), followed by “delaying cannabis use until age 16” (91.2%) and “not driving a car or operating other machinery after using alcohol or other drugs” (88.9%). Among students with lifetime non-medical cannabis use, the average number of recommendations met was 3.8 of the 7 evaluated.

**Table 2:** Lifetime adherence rate for each of the evaluated Lower-Risk Cannabis Use Guidelines, among students reporting lifetime non-medical cannabis use, WMH-ICS survey (N = 9200)

**Guideline**	**Adherence rate among non-medical cannabis users**
Choosing lower-strength cannabis products	29.0%
Not smoking cannabis	36.7%
Never inhaling deeply or holding breath when smoking cannabis	64.2%
Limiting cannabis use as much as possible	78.2%
Not driving a car or operating other machinery after using alcohol or other drugs	88.9%
Delaying use of cannabis until age 16	91.2%
Not using synthetic cannabis	96.1%

**Abbreviation :** WMH-ICS, World Mental Health-International College Student.

### Sociodemographic correlates of Lower-Risk Cannabis Use Guideline adherence

The results of the zero-inflated Poisson models evaluating the correlates of any lifetime cannabis use and the number of unmet LRCUG recommendations among lifetime users are presented in Table 3. The results of the logistic regression models evaluating the correlates of each individual LRCUG are presented in Table 4.

**Table 3:** Multivariable adjusted odds ratios of lifetime non-medical cannabis use and multivariable adjusted incidence ratios for the number of unmet Lower-Risk Cannabis Use Guideline recommendations, WMH-ICS survey

**Characteristics**	**Part 1: Multivariable adjusted ORs of lifetime cannabis use** N = 25 709	**Part 2: Multivariable adjusted IRRs for the number of unmet lower-risk guideline recommendations** N = 8934
**Gender**		
Cisgender man	Reference	Reference
Cisgender woman	0.82 (0.77–0.87)*	0.86 (0.84–0.88)*
Transgender man	0.65 (0.36–1.17)	0.92 (0.77–1.10)
Transgender woman	0.75 (0.31–1.83)	0.65 (0.49–0.87)*
Non-binary	1.10 (0.88–1.38)	0.95 (0.90–1.01)
Two-spirit	1.46 (0.46–4.59)	0.86 (0.61–1.21)
Other	0.93 (0.65–1.32)	0.93 (0.83–1.05)
**Sexual orientation**		
Heterosexual	Reference	Reference
Gay or lesbian	1.74 (1.49–2.02)*	1.01 (0.96–1.06)
Bisexual	3.02 (2.74–3.34)*	1.08 (1.05–1.12)*
Asexual	0.67 (0.51–0.88)*	0.90 (0.80–1.01)
Questioning	1.30 (1.14–1.48)*	1.03 (0.98–1.09)
Other	1.86 (1.53–2.25)*	1.05 (0.98–1.11)
**Race and ethnicity**		
White	Reference	Reference
First Nations, Inuit, or Métis	0.78 (0.57–1.06)	1.14 (1.01–1.29)*
Hispanic or Latino	0.78 (0.63–0.96)*	1.00 (0.93–1.08)
Black	0.41 (0.33–0.51)*	1.09 (1.01–1.17)*
Arab	0.40 (0.32–0.50)*	1.11 (1.02–1.20)*
East Asian (e.g. Chinese, Korean, Japanese)	0.19 (0.18–0.21)*	0.97 (0.93–1.00)*
Southeast Asian (e.g. Filipino, Vietnamese, Cambodian, Laotian, Thai)	0.38 (0.31–0.45)*	1.05 (0.98–1.13)
South Asian (e.g. East Indian, Pakistani, Sri Lankan)	0.36 (0.33–0.40)*	1.06 (1.02–1.10)*
West Asian (e.g. Afghan, Iranian)	0.36 (0.30–0.43)*	1.10 (1.03–1.17)*
Other	0.44 (0.37–0.53)*	0.95 (0.88–1.03)
Multiracial	0.76 (0.69–0.84)*	1.06 (1.03–1.10)*
**Housing type**		
With parents or other relatives	Reference	Reference
In their own home or apartment	1.39 (1.28–1.51)*	1.02 (0.99–1.05)
In a university-owned or -operated residence or fraternity	1.44 (1.29–1.61)*	1.00 (0.96–1.04)
In a shared house, apartment, or flat	1.82 (1.67–1.98)*	1.06 (1.03–1.09)*
Other	1.05 (0.81–1.36)	1.08 (0.98–1.20)
**Student status**		
Domestic	Reference	Reference
International	0.54 (0.49–0.59)*	0.96 (0.93–1.00)*

**Abbreviations:** IRRs, incidence rate ratios; LRCUG, Lower-Risk Cannabis Use Guideline; ORs, odds ratios; WMH-ICS, World Mental Health-International College Student.

**Notes:** Each of the multivariable adjusted odds ratios and multivariable adjusted risk differences are adjusted for all of the other predictors in the table, as well as age.

Incidence ratios for the number of unmet LRCUG were calculated among the sample of lifetime non-medical cannabis users.

* *p* ≤ 0.05.

**Table 4:** Multivariable adjusted odds ratios of not meeting each of the seven Lower-Risk Cannabis Use recommendations evaluated, WMH-ICS survey

**Characteristics**	**Multivariable aOR of initiating cannabis use before age 16**	**Multivariable aOR of not choosing lower-strength cannabis products**	**Multivariable aOR of using synthetic cannabis products**	**Multivariable aOR of smoking cannabis**	**Multivariable aOR of inhaling deeply or holding one’s breath when smoking cannabis**	**Multivariable aOR of not limiting cannabis use to once a week or on weekends**	**Multivariable aOR of driving a car or operating other machinery while under the influence of cannabis**
**Gender**							
Cisgender man	Reference	Reference	Reference	Reference	Reference	Reference	Reference
Cisgender woman	0.63 (0.54–0.74)*	0.69 (0.65–0.74)*	0.66 (0.53–0.83)*	0.74 (0.69–0.79)*	0.71 (0.65–0.77)*	0.53 (0.47–0.58)*	0.50 (0.44–0.58)*
Other (including transgender, non- binary, and two-spirit)	0.95 (0.69–1.30)	0.74 (0.62–0.88)*	0.70 (0.40–1.22)	0.92 (0.77–1.11)	0.95 (0.77–1.16)	0.90 (0.71–1.14)	0.67 (0.46–0.96)
**Sexual orientation**							
Heterosexual	Reference	Reference	Reference	Reference	Reference	Reference	Reference
Gay or lesbian	1.25 (0.87–1.79)	1.53 (1.31–1.78)*	1.90 (1.20–3.02)*	1.45 (1.24–1.69)*	1.63 (1.36–1.95)*	1.37 (1.09–1.73)	0.97 (0.69–1.36)
Bisexual	3.01 (2.51–3.61)*	2.04 (1.87–2.24)*	2.33 (1.74–3.12)*	2.32 (2.11–2.54)*	2.44 (2.19–2.72)*	2.60 (2.28–2.96)*	1.72 (1.41–2.09)*
Asexual	1.16 (0.62–2.17)	0.61 (0.45–0.82)*	1.53 (0.67–3.5)	0.56 (0.41–0.76)*	0.72 (0.50–1.05)	0.45 (0.25–0.83)	0.80 (0.41–1.57)
Questioning	1.88 (1.4–2.52)*	1.20 (1.05–1.38)	1.30 (0.79–2.15)	1.11 (0.96–1.28)	1.28 (1.08–1.53)*	1.52 (1.22–1.89)*	1.43 (1.05–1.93)
Other	2.04 (1.41–2.95)*	1.52 (1.26–1.83)*	1.78 (0.98–3.21)	1.40 (1.15–1.70)*	1.63 (1.31–2.04)*	2.11 (1.63–2.74)*	1.55 (1.06–2.25)
**Race and ethnicity**							
White	Reference	Reference	Reference	Reference	Reference	Reference	Reference
First Nations, Inuit, or Métis	2.59 (1.65–4.07)*	0.79 (0.57–1.10)	1.07 (0.39–2.95)	1.00 (0.73–1.39)	1.11 (0.75–1.65)	1.47 (0.98–2.22)	0.98 (0.54–1.78)
Hispanic or Latino	0.65 (0.38–1.14)	0.97 (0.79–1.20)	0.74 (0.32–1.72)	0.94 (0.76–1.16)	0.91 (0.69–1.21)	0.74 (0.52–1.05)	0.66 (0.39–1.11)
Black	0.24 (0.10–0.57)*	0.66 (0.53–0.82)*	0.40 (0.13–1.28)	0.58 (0.46–0.74)*	0.88 (0.66–1.16)	0.67 (0.46–0.96)	0.53 (0.29–0.95)
Arab	0.53 (0.28–1.01)	0.6 (0.48–0.76)*	0.71 (0.29–1.77)	0.57 (0.45–0.73)*	0.70 (0.51–0.95)	0.96 (0.69–1.34)	0.39 (0.19–0.79)
East Asian (e.g. Chinese, Korean, Japanese)	0.25 (0.20–0.33)*	0.29 (0.26–0.32)*	0.50 (0.36–0.70)*	0.22 (0.20–0.25)*	0.29 (0.26–0.34)*	0.18 (0.15–0.22)*	0.52 (0.42–0.63)*
Southeast Asian (e.g. Filipino, Vietnamese, Cambodian, Laotian, Thai, etc.)	0.37 (0.20–0.67)*	0.57 (0.47–0.69)*	0.92 (0.48–1.76)	0.48 (0.39–0.59)*	0.61 (0.47–0.79)*	0.53 (0.38–0.75)*	0.61 (0.38–1.00)
South Asian (e.g. East Indian, Pakistani, Sri Lankan, etc.)	0.36 (0.27–0.48)*	0.51 (0.46–0.56)*	0.53 (0.35–0.81)*	0.53 (0.48–0.59)*	0.66 (0.58–0.75)*	0.51 (0.43–0.61)*	0.51 (0.40–0.66)*
West Asian (e.g. Afghan, Iranian, etc.)	0.32 (0.17–0.60)*	0.53 (0.44–0.64)*	0.80 (0.41–1.56)	0.59 (0.49–0.71)*	0.82 (0.65–1.04)	0.43 (0.3–0.61)*	0.53 (0.34–0.83)*
Other	0.48 (0.28–0.80)*	0.53 (0.44–0.65)*	0.44 (0.18–1.08)	0.54 (0.44–0.66)*	0.51 (0.39–0.68)*	0.65 (0.48–0.89)*	0.73 (0.48–1.10)
Multiracial	0.86 (0.7–1.07)	0.89 (0.81–0.98)	1.12 (0.81–1.53)	0.95 (0.86–1.04)	1.06 (0.94–1.19)	0.87 (0.75–1.01)	1.02 (0.83–1.25)
**Housing type**							
With parents or other relatives	Reference	Reference	Reference	Reference	Reference	Reference	Reference
In their own home or apartment	1.35 (1.09–1.66)*	1.27 (1.17–1.39)*	1.37 (1.00–1.89)	1.34 (1.23–1.47)*	1.22 (1.09–1.37)*	1.65 (1.43–1.90)*	1.11 (0.93–1.34)
In a university-owned or -operated residence or fraternity	1.15 (0.87–1.51)	1.38 (1.23–1.54)*	1.05 (0.69–1.61)	1.50 (1.34–1.69)*	1.34 (1.17–1.55)*	1.22 (1.00–1.48)	0.83 (0.61–1.13)
In a shared house, apartment, or flat	1.62 (1.32–1.99)*	1.63 (1.49–1.78)*	1.38 (1.01–1.89)	1.85 (1.69–2.03)*	1.66 (1.49–1.85)*	1.69 (1.46–1.94)*	1.20 (0.99–1.46)
Other	1.47 (0.82–2.62)	1.16 (0.90–1.51)	1.01 (0.37–2.72)	1.04 (0.78–1.39)	1.10 (0.78–1.55)	1.53 (1.03–2.28)	1.12 (0.65–1.93)
**Student status**							
Domestic	Reference	Reference	Reference	Reference	Reference	Reference	Reference
International	0.64 (0.5–0.84)*	0.64 (0.58–0.70)*	0.76 (0.53–1.09)	0.63 (0.57–0.69)*	0.51 (0.44–0.58)*	0.62 (0.53–0.74)*	0.56 (0.44–0.71)*

**Abbreviations:** aOR, adjusted odds ratio; WMH-ICS, World Mental Health-International College Student.

**Notes:** The adjusted odds ratios were calculated using multivariable logistic regression.

The multivariable adjusted odds ratios are adjusted for all of the other predictors in the table, as well as age.

* *p* ≤ 0.007 (*p* ≤ 0.05 after Bonferroni correction).

### Gender

Compared to cisgender men, cisgender women had significantly lower odds of lifetime non-medical cannabis use (aOR = 0.82, 95% CI: 0.77–0.87) and, among cannabis users, a lower incidence of LRCUG recommendation non-adherence (aIRR = 0.86, 95% CI: 0.84–0.88). The largest disparities were in the odds “driving a car or operating machinery with cannabis use” (aOR = 0.5, 95% CI: 0.44–0.58) and “not limiting cannabis use” (aOR = 0.53, 95% CI: 0.47–0.58).

### Sexual orientation

With the exception of asexual students, non-heterosexual students indicated significantly higher odds of lifetime non-medical cannabis use, with bisexual students reporting the highest odds (aOR = 3.02, 95% CI: 2.74–3.34). Conversely, asexual students reported significantly lower odds of lifetime non-medical cannabis use (aOR = 0.67, 95% CI: 0.51–0.88). Among lifetime cannabis users, bisexual students (aIRR = 1.08, 95% CI: 1.05–1.12) reported a higher incidence of LRCUG recommendation non-adherence than heterosexual students, though the effect was small. The extent of non-adherence of individual LRCUG recommendations was heterogeneous across non-heterosexual student groups, though highest in bisexual students across guidelines. In particular, bisexual students reported 3.01 times the odds of initiating cannabis use before the age of 16 (aOR = 3.01, 95% CI: 2.51–3.61).

### Race and ethnicity

Racial and ethnic minority students reported lower odds of lifetime non-medical cannabis use compared to White students, with East Asian students reporting the lowest odds of lifetime cannabis use (aOR = 0.19, 95% CI: 0.18–0.21), followed by West Asian (aOR = 0.36, 95% CI: 0.30–0.43), South Asian (aOR = 0.36, 95% CI: 0.33–0.40), and Southeast Asian (aOR = 0.38, 95% CI: 0.31–0.45) students. Conversely, among cannabis users, most racial and ethnic minority groups reported a higher incidence of LRCUG recommendation non-adherence, with First Nations, Inuit, and Métis students reporting the highest incidence of LRCUG recommendation non-adherence (aIRR = 1.14, 95% CI: 1.01–1.29), followed by Arab (aIRR = 1.11, 1.02–1.20) and West Asian (aIRR = 1.10, 95% CI: 1.03–1.17) students. East Asian students reported a lower incidence of LRCUG recommendation non-adherence among lifetime cannabis users, though the effect was small (aIRR = 0.97, 95% CI: 0.93–1.00). Compared with White students, First Nations, Inuit, or Métis students reported more than double the odds of initiating cannabis use before age 16 (aOR = 2.59, 95% CI: 1.65–4.07).

### Housing

Students living on their own reported higher odds of lifetime non-medical cannabis use than students living with their parents or other relatives, with students living in a shared house, apartment, or flat (aOR = 1.82, 95% CI: 1.67–1.98) reporting the highest odds. In particular, students living in a shared house, apartment, or flat had 1.85 times the odds of smoking cannabis (aOR = 1.85, 95% CI: 1.69–2.03), compared to students living with family. Additionally, all groups of students living apart from their parents or other relatives reported higher odds of not meeting the “limiting cannabis use to once a week or weekends” recommendation, with the students living in a shared house, apartment, or flat reporting the highest odds of guideline non-adherence (1.69, 95% CI: 1.46–1.94).

### International student status

International students reported significantly lower odds than domestic students (aOR = 0.54, 95% CI: 0.49–0.59) and a slightly lower incidence of LRCUG recommendation non-adherence among lifetime users (aIRR = 0.96, 95% CI: 0.93–1.00). International students reported the highest odds of following each of the individual recommendations, with the lowest comparative odds of inhaling deeply or holding their breath when smoking cannabis (aOR = 0.51, 95% CI: 0.44–0.58) and driving or operating other machinery while under the influence (aOR = 0.56, 95% CI: 0.44–0.71).

The results of the corresponding univariable, unadjusted analyses are available upon request from the authors.

## Discussion

### Key findings

This study evaluated the prevalence of cannabis use and adherence to LRCUG recommendations among university students, as well as the sociodemographic differences in LRCUG adherence. Optimistically, most students (66.2%) did not use cannabis, and among cannabis users, there were high rates of avoiding synthetic cannabis (96.1%), delaying cannabis use until age 16 (91.2%), not driving a car or operating other machinery after using alcohol or other drugs (88.9%), and limiting cannabis use as much as possible (78.2%). However, there were low rates of not smoking cannabis (36.7%) and choosing lower-strength cannabis products (29.0%) among users. The Canadian Postsecondary Education Alcohol and Drug Use Survey (CPADS) and Canadian Cannabis Survey (CCS) reported higher rates of cannabis use among students, with CPADS reporting a 12-month prevalence of 39.5% and CCS reporting a 12-month prevalence of 39.2%.^28^^,^^29^ These differences may reflect the weighting of CPADS and CCS to reflect sex/age breakdowns, as well as the academic rigour of the Canadian universities surveyed in this study; research has shown that frequent cannabis use predicts a lower likelihood of postsecondary enrolment and degree attainment.^28^^,^^29^ The patterns of guideline adherence among cannabis users generally align with those of the broader population; the 2018 International Cannabis Policy Study also reported smoking cannabis and use of high-strength cannabis as the guidelines most commonly unmet among Canadians and Americans aged 16 to 65.^18^

The low rates of choosing lower-strength cannabis products may be, in part, attributable to lack of knowledge. Among lifetime cannabis users in the study sample, 18.2% were aware of the existence of THC and CBD but not aware of their different properties and 2.7% were not aware of their existence altogether. Users with lesser knowledge about THC/CBD were significantly less likely to follow the “choosing lower-strength cannabis products” guideline. Similar findings have been reported among Canadian and American adults, with low proportions of 12-month cannabis users using products with equal or higher CBD levels, and a substantial proportion reporting that they did not know the relative levels in the products they typically used.^31^ It is important to note the low availability of lower-potency cannabis products available; a 2022 analysis of Ontario Cannabis Store offerings found that the vast majority of inhalation products (94%–100%) had a high THC concentration.^32^ These findings add to the mounting evidence highlighting the need for education conveying the nuances of lower-risk cannabis use, as well as availability of lower-risk cannabis products. Of note, Wood et al. recently proposed a simplified product labelling system based on “Canadian THC Units” that could be integrated into future LRCUG guidelines and cannabis product labels to enable cannabis users to more easily and accurately assess their THC dosing levels across different product types and modes of administration.^33^

Men, non-heterosexual students, students living in shared housing, and domestic students were more likely to use cannabis and, among users, reported riskier use. White students were most likely to use cannabis; however, among users, non-White student groups (with the exception of East Asian and “other” students), reported riskier use. These findings generally align with the literature. For example, the more common, intensive, and riskier use in men has been well documented and may be shaped by gender norms.^34^ Additionally, the CCS also reported a high prevalence of cannabis use in sexual and gender minority (SGM) individuals, which is posited to arise from its role in coping with societal stressors and the openness in SGM communities.^29^^,^^35^ The CPADS also reported the highest rates of cannabis use in Indigenous, multiracial, and White postsecondary students.^28^ Interestingly, among American youth, Black, Indigenous, Hispanic and Asian American past-year users reported higher odds of cannabis use disorder than their White counterparts, further confirming the trend in our data and highlighting the importance of targeting the subpopulation of racial/ethnic minority users.^36^

### Strengths and limitations

There are several key strengths and limitations of this study. This appears to be the first study of adherence to LRCUG recommendations among university students in Canada. Understanding rates of adherence to LRCUG recommendations and associated sociodemographic correlates in this population provides important insights to inform targeted intervention and prevention initiatives in a population where both cannabis use and hazardous cannabis use are common.^30^ A major methodological strength of the repeated cross-sectional survey design was the use of weekly stratified random sampling (by gender, age, degree type and year, and international student status) to recruit a large sample of students. Additionally, efforts were made to reduce non-responder bias through use of a multistage recruitment strategy.^25^ There are also several limitations to be considered. Firstly, while our stratified random sampling strategy provided a representative sample of survey invitees, our sample was not weighted to improve representativeness of respondents. Additionally, the assessment of cannabis use was limited to any lifetime use which prevented the assessment of both frequency and recency of use. For some, “lifetime use” may simply represent one-time use. Further, our study examined adherence to the 2017 LRCUG as opposed to the most updated 2022 version, since our data was collected from 2020 to 2022. While the 2017 LRCUG remain cited by Health Canada and largely overlap with the 2022 LRCUG, one key recommendation we did not evaluate was the avoidance of vaping.^17^ The 2022 LRCUG acknowledge that vaping has a lower level of toxin exposure than smoking, but emphasize that it still carries the risks associated with cannabis inhalation and potential exposure to other contaminants, making it an important behaviour for future study.^8^ Lastly, the generalizability of the results of this study is limited by the four universities from which students were recruited; the sample does not include data from the numerous colleges and smaller universities across Canada which have been shown to have higher rates of cannabis use than those found in this study.

## Conclusion

To our knowledge, this is the first study to examine adherence to the LRCUG recommendations among Canadian university students—a distinct population with opportunities for targeted public health initiatives. While our findings reveal several encouraging patterns of harm reduction, they also highlight critical gaps in LRCUG recommendation adherence, particularly regarding methods of consumption and cannabis product potency. Although some of these behaviours may reflect personal preference, our study indicates that significant knowledge gaps persist. Future interventions should aim to directly address these gaps. In the university setting, this could include orientation events, campus posters, and messaging through student health and wellness services. Campus residences, with their centralized structure and built-in communication networks, also offer an ideal environment for knowledge translation. Adoption of a standard THC unit on cannabis labels and increased availability of lower-potency cannabis products is also needed to facilitate low-risk use.

## Data Availability

None.
